# New Books

**Published:** 2008-02

25 Years of P53 Research

Wiman, Klas G., Hainaut, Pierre, eds.

New York:Springer, 2008. 448 pp. ISBN: 978-1-4020-6564-4, $69.95

Advances in Molecular Toxicology, 2

James C. Fishbein

Burlington, MA:Elsevier, 2008. 275 pp. ISBN: 978-0-444-53098-1, $199.95

Biosecurity and Bioterrorism

Jeffrey Ryan, Jan Giarum

Burlington, MA:Elsevier, 2008. 352 pp. ISBN: 978-0-7506-8489-7, $69.95

Cancer Proteomics: From Bench to Bedside

Sayed S. Daoud, ed.

New York:Springer, 2008. 300 pp. ISBN: 978-1-58829-858-4, $125

Class 2 Transferases XII

Dietmar Schomburg, Ida Schomburg, A. Chang, eds.

New York:Springer, 2008. 550 pp. ISBN: 978-3-540-71523-8, $389

Environmental Crises

Hans von Storch, Richard Tol, Götz Flöser, eds.

New York:Springer, 2008. 146 pp. ISBN: 978-3-540-75895-2, $109

Environmental Policy Instruments for Conserving Global Biodiversity

Oliver Deke

New York:Springer, 2008. 392 pp. ISBN: 978-3-540-73747-6, $149

Environmental Regulatory Calculations Handbook

Louis Theodore

Hoboken, NJ:John Wiley & Sons, Inc., 2007. 561 pp. ISBN: 978-0-471-67171-8, $145

Globalization and Environmental Challenges: Reconceptualizing Security in the 21st Century

H.G. Brauch, Spring, Ú. Oswald, C. Mesjasz, J. Grin, P. Dunay, N.C. Behera, B. Chourou, P. Kameri-Mbote, P.H. Liotta, eds.

New York:Springer, 2008. 1,148 pp. ISBN: 978-3-540-75976-8, $309

Global Risk Governance: Concept and Practice Using the IRGC Framework

O. Renn, K. Walker, eds.

New York:Springer, 2008. 370 pp. ISBN: 978-1-4020-6798-3, $149

Metabolomics

Jens Nielsen, Michael C. Jewett, eds.

New York:Springer, 2007. 284 pp. ISBN: 978-3-540-74718-5, $189

Oil and Security: A World beyond Petroleum

E.G. Frankel

New York:Springer, 2008. 184 pp. ISBN: 978-1-4020-6381-7, $119

Peptidomics: Methods and Applications

Mikhail Soloviev, Per Andrén, Chris Shaw

Hoboken, NJ:John Wiley & Sons, Inc., 2007. 401 pp. ISBN: 978-0-471-67781-9, $99.95

Practical Bioinformatics

Janusz M. Bujnicki, ed.

New York:Springer, 2008. 265 pp. ISBN: 978-3-540-74267-8, $99

Review of the U.S. Climate Change Science Program’s Draft Synthesis and Assessment Product 2.4: Trends in Emissions of Ozone Depleting Substances, Ozone Layer Recovery, and Implications for Ultraviolet Radiation Exposure

Committee to Review the U.S. Climate Change Science Program’s Draft Synthesis and Assessment Product 2.4, National Research Council

Washington, DC:National Academies Press, 2007. 76 pp. ISBN: 978-0-309-11525-4, $18.90

Risk Assessment for Chemicals in Drinking Water

Robert A. Howd, Anna M. Fan

Hoboken, NJ:John Wiley & Sons, Inc., 2007. 392 pp. ISBN: 978-0-471-72344-8, $99.95

The Black Sea Environment

Andrey G. Kostianoy, Aleksey N. Kosarev, eds.

New York:Springer, 2008. 457 pp. ISBN: 978-3-540-74291-3, $329

The Joy of Science

Richard A. Lockshin

New York, NY: Springer, 2008, 450 pp. ISBN: 978-1-4020-6098-4, $89.95

